# Seroprevalence of *Entamoeba histolytica* Infection among Chinese Men Who Have Sex with Men

**DOI:** 10.1371/journal.pntd.0002232

**Published:** 2013-05-23

**Authors:** Feng Zhou, Mufei Li, Xiangwei Li, Yu Yang, Cong Gao, Qi Jin, Lei Gao

**Affiliations:** MOH Key Laboratory of Systems Biology of Pathogens, Institute of Pathogen Biology, Chinese Academy of Medical Sciences & Peking Union Medical College, Beijing, China; George Washington University, United States of America

## Abstract

**Background:**

Men who have sex with men (MSM) were found to be one of the high-risk populations for *Entamoeba histolytica* (*E. histolytica*) infection. Accompanied by the prevalence of human immunodeficiency virus (HIV) among MSM, invasive amebiasis caused by *E. histolytica* has been paid attention to as an opportunistic parasitic infection. However, the status of *E. histolytica* infection among MSM has been barely studied in mainland China.

**Methods:**

Seroprevalance of *E. histolytica* was determined using an enzyme-linked immunosorbent assay based on a cross-sectional study conducted in Beijing and Tianjin, China. Factors potentially associated with *E. histolytica* infection were identified by logistic regression analysis.

**Results:**

A total of 602 MSM were included in the study and the laboratory data on serostatus of *E. histolytica* were available for 599 of them (99.5%). 246 (41.1%) and 51 (8.5%) of the study participants were *E. histolytica* seropositive and HIV seropositive, respectively. Univariate analyses suggested preferred anal sex behaviors were associated with *E. histolytica* seropositivity. In multivariate logistic regression analysis, “only has receptive anal sex” (OR: 2.03; 95% CI: 1.22 3.37), “majority receptive anal sex” (OR: 1.83; 95% CI: 1.13, 2.95), and “sadomasochistic behavior (SM)” (OR: 2.30; 95% CI: 1.04, 5.13) were found to be significantly associated with *E. histolytica* infection.

**Conclusions:**

High seroprevalence of *E. histolytica* infection was observed among MSM from Beijing and Tianjin, China. Receptive anal sex behavior and SM were identified as potential predictors. Therefore, *E. histolytica* and HIV co-infection needs to be concerned among MSM due to their sharing the common risk behaviors.

## Author Summary

*Entamoeba histolytica* (*E. histolytica*) is a very common human gastrointestinal parasitic disease which affects 50 million people worldwide. Men who have sex with men (MSM) have already been found to be one of the high-risk populations with *E. histolytica* infection. Previous studies have reported an increased risk for *E. histolytica* infection and invasive amebiasis in HIV seropositive MSM. This pilot study aimed to investigate the serology of *E. histolytica* among MSM from mainland China. High prevalence of *E. histolytica* infection (41.1%, 246/599) was observed among the study population, receptive anal sex behavior and sadomasochistic behavior were found to be associated with the *E. histolytica* serostatus. Although HIV infection was not found to be associated with *E. histolytica* infection in this pilot study, studies from other countries had reported increased risks for *E. histolytica* infection and invasive amebiasis among HIV-positive MSM. Our findings suggest *E. histolytica* infection control needs to be concerned with respect to the increasing HIV prevalence among Chinese MSM population.

## Introduction

*Entamoeba histolytica* (*E. histolytica*) has a worldwide distribution and is endemic in most developing countries. Invasive amebiasis (IA) caused by *E. histolytica* is a very common human gastrointestinal parasitic disease which affected 50 million people worldwide and caused greater than 100,000 deaths annually. High risk populations for developing IA include infants, travelers from endemic area, and patients who are taking immunosuppressant [1], [2]. In mainland China, *E. histolytica* infection was also very popular in general population. The average prevalence of *E. histolytica* infection was 0.95%, ranged from 0.01% to 8.12% [3].

In 1967, the association between amebiasis and homosexuality was suggested for the first time [4]. Men who have sex with men (MSM) population had already been found to be a high risk population with *E. histolytica* infection before 1990. Homosexuality and oral-anal sex have been most frequently reported as potential risk factors for *E. Histolytica* infection [5]–[13]. Accompanied by the transmission of human immunodeficiency virus (HIV) in MSM population, the prevalence of IA caused by *E. histolytica* are increasing and getting the attention as an important opportunistic parasitic infection. Recent studies from Australia, Japan, Korea and Taiwan reported increased risks for *E. histolytica* infection and IA among HIV-positive MSM [14]–[18]. Hung CC and colleagues recently reviewed the status of *E. Histolytica* infection in MSM [19]. By the end of 2011, China had about 780,000 people living with HIV/AIDS and 17.4% of them were MSM. The estimated new HIV infections in 2011 are 48,000 and 38.1% were MSM [20]. Case report of IA suggested the risk of *E. histolytica* prevalence among Chinese MSM, especially in the HIV positives [21]. However, to our knowledge, the prevalence of *E. histolytica* infection among MSM population has not been investigated in mainland China. The major aim of this study was to assess the seroprevalence of *E. histolytica* infection and the potential impact factors among MSM from China.

## Methods

### Ethics statement

The study was approved by the Ethics Committees of the Institute of Pathogen Biology, Chinese Academy of Medical Sciences & Peking Union Medical College. All participants were the adults of men who had sex with men. Written informed consent was obtained before the interview and testing.

### Study design and participants

A cross-sectional study was conducted in Beijing and Tianjin, China. Six hundred study participants were recruited between March and July 2010, though local non-government organizations (Beijing Rainbow Volunteers Workstation and Tianjin Deep Blue Volunteers Workgroup). Participants' recruitment and inclusion criterion had been addressed in previous study [22].

### Data collection

Questionnaire administered by the trained interviewer were performed for each study participant, the data acquired from the questionnaire includes socio-demographic characteristics (e.g., age, income, ethnicity, education, employed, and marital status), sexual orientation and homosexual act, sexual behaviors in the past 6 months, history of STDs and HIV infection. Anilingus behaviors were defined as sexual stimulation involving oral contact with the anus and sadomasochistic behaviors (SM) were defined as behaviors which aimed to enhance sexual gratification from inflicting or submitting to physical and emotional abuse.

### Sample collection and laboratory tests

Blood samples were collected for *E. histolytica* and HIV serology. Serum samples were stored at −80°C until tested. Each study participant was assigned a unique identification number that was used to link the questionnaire and specimens. The HIV infection status was screened by an enzyme immunoassay (Wantai Biological Medicine Company, Beijing, China), and positive tests were confirmed by HIV-1/2 Western blot assay (HIVBlot 2.2 WB; Genelabs Diagnostics, Singapore). Qualitative screenings of serum immunoglobulin G (IgG) antibodies to *E. histolytica* were retrospectively performed using the commercial enzyme-linked immunosorbent assay (ELISA) kit (Shanghai Fengxiang Biological Technology Co. Ltd. Shanghai, China). Purified *E. histolytica* antigen was used to coat microtitration wells, incubated for 30 minutes at 37°C after adding 10 uL serum samples to wells. Washing and removing non-combinative antibody and other components, then combined HRP-conjugate reagent, then incubated and washed again. The substrate solution was added to each well, after 15 minutes at 37°C, stop solution was added to arrest color development and a ELISA reader was used to measure the absorbance at 450 nm. Each sample was tested in duplicate and the average optical density (OD) value was calculated. Test validity was evaluated as: the average of the positive controls should ≥1.00 and the average of negative controls should ≤0.10. The cut off value was set as the average of negative controls +0.15 according to the instruction of the kit.

### Statistical analysis

Questionnaires were double entered and compared with EpiData software (EpiData 3.02 for Windows, The Epi Data Association Odense, Denmark). After cleaning, the data were then converted and analyzed using Statistical Analysis System (SAS 9.2 for Windows; SAS Institute Inc., NC, USA).

Study population was characterized by site with respect to age, ethnicity, education, marriage status, and current status of HIV. Differences between sites in these variables were assessed with Pearson chi-square test. The associations of *E. histolytica* infection with the characteristics of demographics, sexual behaviors, diagnosed STDs including HIV, and current status of HIV were estimated using Pearson chi-square test. Variables related with *E. histolytica* serology (p<0.1) in the univariate analyses were included in a multiple logistic regression model additionally adjusted for age, site, and HIV infection status. The Cochran-Armitage test was used to evaluate the trend of OD value in *E. histolytica* antibody test associated with anal sex behaviors categories.

## Results

### Demographic characteristics and its association with *E. histolytica* infection

A total of 607 participants were interviewed and signed the informed consent, 6 of them were excluded (3 did not complete sample collection and 2 Vietnamese living in Beijing). Finally, 602 were used for the analyses (302 from Beijing and 300 from Tianjin). Laboratory data on serostatus of *E. histolytica* and HIV were available for 599 (99.5%) and 598 (99.3%) of the study participants respectively. Age, ethnicity, education, marriage status, HIV-1 serostatus and *E. histolytica* serostatus of the study population were compared by site (Table 1). No significant difference was found for any characters (p>0.05). Therefore, participants from the two sites were pooled together for further association analyses. Age of the participants ranged from 16 to 72 years, with a mean age of 27.9±8.3 years. The participants showed the following characteristics: 95% (570/600) were Han nationality; 51.5% (309/600) had more than 12 year's education; 77.7% (467/601) were single. Laboratory data suggested two hundred and forty six (41.1%) and fifty one (8.5%) of the study participants were *E. histolytica* seropositive and HIV seropositive respectively. Twenty three participants were *E. histolytica* and HIV co-infected. Age, ethnicity, site, registered residence, education, marriage status, and alcohol use were not found to be related with *E. histolytica* serostatus (Table 2).

**Table 1:** Characteristics of the study population by study site.

Factors	Beijing (N = 302)	Tianjin (N = 300)	p for difference
	n* (%)	n* (%)	
**Age**			
≤19 years	17 (6.0)	18 (6.3)	
20–29 years	184 (60.9)	199 (66.3)	
30–39 years	66 (21.9)	45 (15.0)	
≥40 years	34 (11.3)	37 (12.3)	0.194
**Ethnicity**			
Han	285 (94.7)	285 (95.3)	
Others	16 (5.3)	14 (4.7)	0.722
**Education**			
≤9 years	64 (21.3)	44 (14.7)	
10–12 years	93 (31.0)	90 (30.0)	
>12 years	143 (47.7)	166 (55.3)	0.065
**Marriage status**			
Unmarried	232 (77.1)	235 (78.3)	
Married/divorced/widowed	69 (22.9)	65 (21.7)	0.711
**HIV-1 serostatus**			
Negative	268 (89.9)	279 (93.0)	
Positive	30 (10.1)	21 (7.0)	0.179
**Entamoeba histolytica serostatus**			
Negative	181 (60.1)	172 (57.7)	
Positive	120 (39.9)	126 (42.3)	0.548

Abbreviation: HIV, human immunodeficiency virus.

* Sum may not always add up to total because of missing data.

**Table 2:** Associations between demographical characteristics and *E. histolytica* serostatus.

Factors	*E. histolytica* Seropositivity n/N* (%)	p for difference
Age		
≤19 years	13/35 (37.1)	
20–29 years	166/381 (43.6)	
30–39 years	42/111 (37.8)	
≥40 years	25/70 (35.7)	0.474
Han Ethnic		
Not	14/29 (48.3)	
Yes	232/568 (40.9)	0.428
Site		
Beijing	120/301(39.9)	
Tianjin	126/298(42.3)	0.548
Registered residence		
Not	93/245 (38.0)	
Yes	153/353 (43.3)	0.188
Education		
≤9 years	63/45 (41.7)	
10–12 years	68/183 (37.2)	
>12 years	133/306 (43.5)	0.388
Marital status		
Single	192/464 (41.4)	
Married/divorced/widowed	54/134 (40.3)	0.823
Alcohol use		
No	166/404 (41.1)	
Yes	80/194 (41.2)	0.973

* Sum may not always add up to total because of missing data.

### Sexual behaviors and its association with *E. histolytica* infection

Homosexual men accounted for 71.0% (427/602), and bisexual men for 24.3% (146/602) of the study population. 73.6% (181/246) of participants with *E. histolytica* seropositive were homosexuals. Age of the first homosexual act ranged from 5 to 55 years old, with a mean age of 21.4±5.0 years. The median number of their homosexual partners was eleven and 43.1% of participants had stable homosexual partners before baseline survey. In the past one year, sixty one participants (10.2%) had group sex, thirty eight participants (6.3%) had ever received money for sex with male partners, and twenty three (3.8%) had ever provided money for sex with male partners. In the past 6 months, 133 (22.2%) participants reported had sexual behavior less than 1 time per month and only 1.3% (8/602) of participants insisted on using condoms in the process of insertive or receptive anal sex and oral sex. 22.5% of participants had ever been diagnosed sexually transmitted diseases. The association between sex behaviors and *E. histolytica* infection were also assessed. Self-reported preferred anal sex behaviors were classified to four types (only has insertive anal sex, majority insertive anal sex, majority receptive anal sex, and only has receptive anal sex). Univariate analyses suggested preferred anal sex behaviors were associated with *E. histolytica* infection. HIV infection was not found associated with *E. histolytica* infection (Table 3).

**Table 3:** Associations between sexual behaviors and *E. histolytica* serostatus.

Factors	*E. histolytica* Seropositivity n/N* (%)	p for difference
Self-reported sexual orientation		
Homosexual	181/424 (42.7)	
Bisexual or uncertain	65/173 (37.6)	0.249
Age at the first homosexual act		
<18 years	31/71 (43.7)	
≥18 years	215/526 (40.9)	0.654
Seeking sexual partners in gay venues		
No	120/265 (45.3)	
Yes	126/333 (37.8)	0.066
Total number of MSM sex partners		
≤10	123/297 (41.4)	
>10	123/301 (40.9)	0.891
Frequency of sexual behavior in the past 6 months		
≤1 per month	61/133 (45.9)	
>1 per month	185/463 (40.0)	0.223
Preferred anal sex behavior		
Insertive only	45/147 (30.6)	
majority insertive	46/120 (38.3)	
majority receptive	77/169 (45.6)	
Receptive only	71/147 (48.3)	0.009
Is oral sex a common sexual behavior?		
Not	71/166 (42.8)	
Yes	175/432 (40.5)	0.615
Is anilingus a common sexual behavior?		
Not	168/415 (40.5)	
Yes	78/183 (42.6)	0.624
SM		
No	229/568 (40.3)	
Yes	17/30 (56.7)	0.076
Anal sex in the past 6 months		
No	22/41 (53.7)	
Yes	224/555 (40.4)	0.095
Consistent condom use in all style of homosexual behaviors in the past 6 months		
No	217/527 (41.2)	
Yes	3/7 (42.9)	0.929
Group sex		
No	215/534 (40.3)	
Yes	29/61 (47.5)	0.274
Having received money for sex with males		
No	227/552 (41.1)	
Yes	14/37 (37.8)	0.694
Having paid sex with males		
No	233/567 (41.1)	
Yes	8/22 (36.4)	0.658
Ever had STDs including HIV-1		
No	184/454 (40.5)	
Yes	56/133 (42.1)	0.745
HIV-1 infection		
No	221/545 (40.6)	
Yes	23/50 (46.0)	0.453
Heterosexual behaviors in the past 6 months		
No	164/375 (43.7)	
Yes	82/223 (36.8)	0.094
Had ever been circumcised		
No	206/503 (41.0)	
Yes	28/57 (49.1)	0.236

Abbreviation: SM, sadomasochism; STDs, sexually transmitted diseases.

* Sum may not always add up to total because of missing data.

The variables associated with *E. histolytica* infection in the univariate analyses (P<0.1) were included in a multivariate logistic regression model. Age, site, and HIV infection status were fixed in the model as well. In the multivariate logistic regression model, only has receptive anal sex (OR: 2.03; 95% CI: 1.22, 3.37), majority receptive anal sex (OR: 1.83; 95% CI: 1.13, 2.95), and SM (OR: 2.30; 95% CI: 1.04, 5.13) were found to be significantly associated with *E. histolytica* infection after adjusted for the other variables (Table 4).

**Table 4:** The associations of *E. histolytica* infection with potential factors in multivariate logistic regression model.

Factors	Ajusted OR (95% CI) *	p value
Prefered anal sex behavior		
Insertive only	Ref.	
Majority insertive	1.43 (0.85, 2.40)	0.174
Majority receptive	1.83 (1.13, 2.95)	0.014
Receptive only	2.03 (1.22, 3.37)	0.006
Seeking sexual partners in gay venues		
No	Ref.	
Yes	0.74 (0.50, 1.08)	0.115
Anal sex in the past 6 months		
No	Ref.	
Yes	0.52 (0.25, 1.07)	0.075
SM*		
No	Ref.	
Yes	2.30 (1.04, 5.13)	0.041
Heterosexual behaviors in the past 6 months		
No	Ref.	
Yes	0.93 (0.63, 1.38)	0.713

Abbreviation: CI, confidential interval; OR, odds ratio; SM, sadomasochism. Anal sex in the past 6 months, preferred anal sex behavior, SM, seeking sexual partners in gay venues and heterosexual behaviors in the past 6 months were included in a multivariate logistic regression model, adjusted by age, site, and HIV infection status.

**Figure 1:**
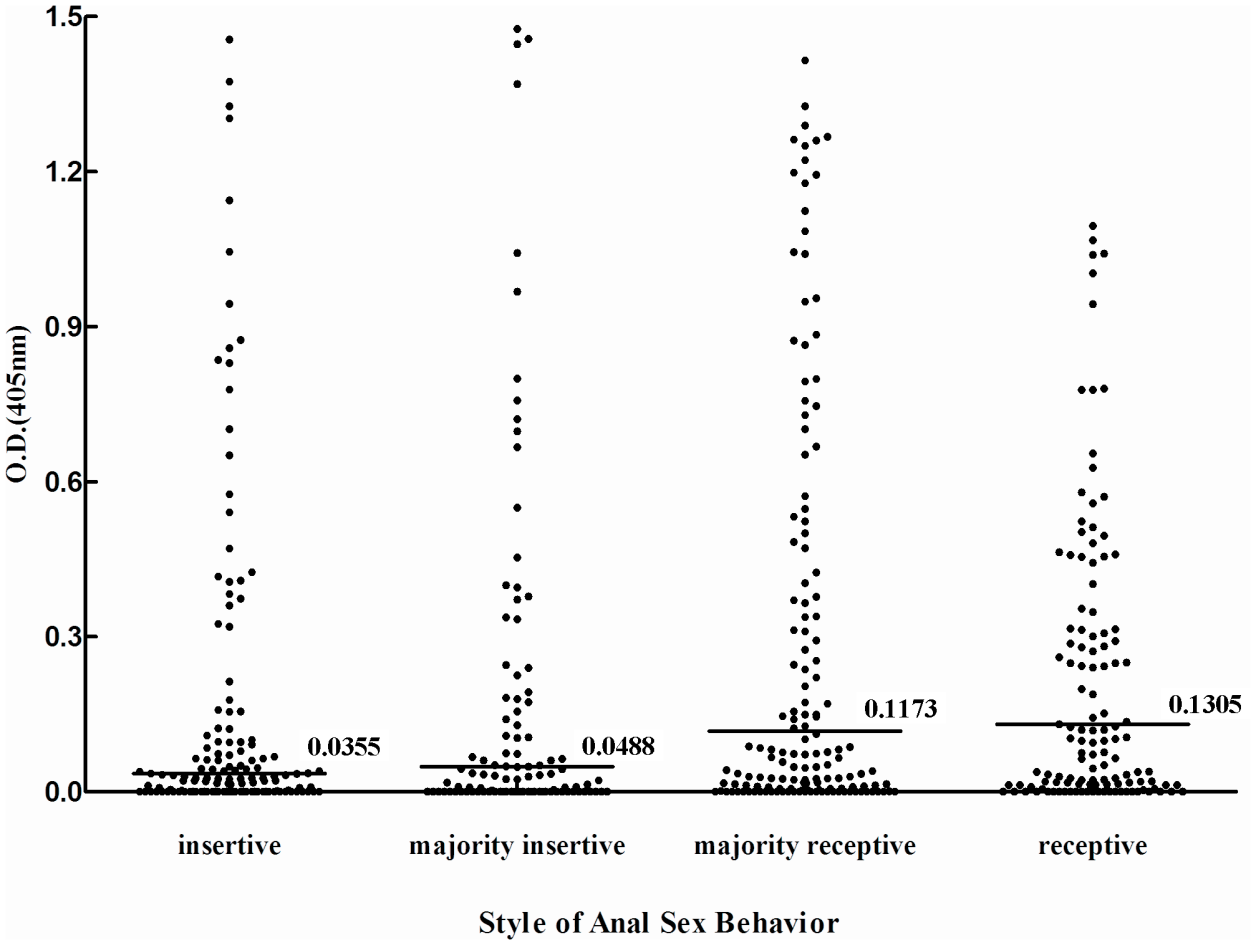
ELISA response for various style of anal sex behavior. Solid horizontal lines and numbers represent median values obtained for each group.

The OD in ELISA test was used for further analysis. Five hundred and eighty three participants reported their preferred anal sex behavior. As shown in figure 1, the OD values increased from the group of only had insertive anal sex (median OD, 0.036), majority insertive anal sex (median OD, 0.049), majority receptive anal sex (median OD, 0.117) to only had receptive anal sex (median OD, 0.131). The trend of increasing was significant (p<0.001), which was consistent with the result of multivariate analysis.

## Discussion

This pilot study investigated *E. histolytica* seroprevelance in MSM from China; potential factors associated with *E. histolytica* infection were evaluated as well. In a total of 602 study participants, *E. histolytica* seroprositivity was found to be 41.1%. Types of preferred anal sex behavior (only has receptive anal sex and majority receptive anal sex) and SM were identified as significant predictors for *E. histolytica* infection. In addition, significant different antibody levels were observed between subgroups with respect to the preferred anal sex behavior.

The first observation of a relation between enteric protozoan infections and sexual behavior was reported in 1968 [4]. Epidemiological studies conducted in the developed countries showed homosexuals or MSM had significant higher risk of *E. histolytica* infection. Using microscopy for diagnosis, the prevalence varied from 20% to 32% among MSM without gastrointestinal symptoms [5], [7], [8], [23]–[27]. However, microscopy is not sensitive or specific enough for the detection of *E. histolytica* in clinical specimens, especially for the differentiation *E. histolytica* from *E dispar and E moshkovskii* in the epidemiological studies with a large sample size. Therefore, serological tests were used to detect the infection though measuring anti-*E. histolytica* antibodies and seroprevalence ranging from 0.2% to 21% using ELISA test among HIV negative MSM were reported in several developed countries [18], [19]. Our results, for the first time, suggested a high prevalence (41.1%) of *E. histolytica* infection among MSM community from China. A recently published study conducted among general population in seven provinces in China showed that the seroprevalence of *E. histolytica* infection varied from 6% to 11% [28]. Although antibody test could not distinguish the past or current infection status and maybe overestimated the epidemic status, the fact that amebic liver abscess and latent infection had become one of the common opportunistic infection diseases among Chinese MSM AIDS patients reminds us to pay attention to the co-infection of *E. histolytica* and HIV. [21], [29].

In the present study, homosexual behaviors were mostly classified according to participants' tendency. Receptive anal sex behavior was found to be related to higher prevalence of *E. histolytica* infection. This finding and its underlying mechanisms should be further studied in the future. Homosexuals and history of anilingus had been demonstrated to be the risk factors of *E. histolytica* infection. In 1978, a study from the New York city reported that 20% of eighty nine sexually active homosexual men had amebiasis and the presence of infections associated with history of anilingus [5]. Another study from a venereal-disease-clinic population compared the prevalence of *E. histolytica* infections in homosexual men, bisexual men and heterosexual men. Homosexuality and oral-anal sex were found to be the most important risk factors for *E. histolytica* infection [7]. However, such an association was not observed in our study population. Interestingly, SM was found to be associated with *E. histolytica* infection in the present study. The data on the specific behaviors during the process of SM has not been well studied in China due to the potential issues of social culture and discrimination. Several published studies had revealed that people who had engaged in SM were more likely to have experienced oral-anal sex and other sexual risk practice [30]–[32]. In addition, fecal-oral contamination in these sexual behaviors maybe occurs and increases the opportunity of pathogen infection. Keystone JS' study showed cleaning of anus before anal sex was associated with a significant lower prevalence of infection [23]. But it is difficult to explore the factors linked SM behaviors to the infection susceptibility in our present study because only 30 participants (5.0%) reported SM behaviors. Developing targeted prevention and control strategies, such as developing sanitary habit before sexual behavior, may decrease the opportunity of pathogen infection. Therefore, it is necessary to further study potential risky behaviors associated with health problems among Chinese MSM.

Several limitations of this study should be kept in mind. First, potential bias due to the inaccurate response to the questionnaire, especially to the questions on sexual behaviors, could not be excluded completely. Second, our study participants might not represent the general MSM population due to the potential limitation of enrollment methods. Therefore, potential selection bias should be considered when interpret our results. Third, serology could not clearly identify the infection status as current infection or past infection, potential bias caused by such misclassification could not be excluded. Although statistically significant difference of antibodies levels was observed with respect to the preferred anal sex behaviors (p<0.001), however, the smaller sample size in each subgroups and a broad range of OD value should be considered. Further studies are needed to explore the underlying mechanisms for the observed relation between receptive anal sex behaviors and *E. histolytica* infection. Fourth, cross-sectional study design has its limitation on association analysis. Therefore, our results need confirmation by further large-scale case-control studies or prospective studies.

In conclusion, high prevalence of *E. histolytica* infection was observed among MSM from Beijing and Tianjin, China. Receptive anal sex behaviors and SM were identified as significant predictors for *E. histolytica* infection. Prevention and control of *E. histolytica* infection among Chinese MSM should be concerned because this special population confronted with high risk of HIV infection.

## Supporting Information

Checklist S1 STROBE Checklist.
(DOC)
